# Pain attenuation during task: a proof-of-concept study for a new scoring metric to capture individual differences in chronic pain changes by task engagement

**DOI:** 10.3389/fpain.2026.1945745

**Published:** 2026-10-26

**Authors:** Vaidhehi Veena Sanmugananthan, Rima El-Sayed, Robert D. Inman, Karen Deborah Davis

**Affiliations:** 1 Institute of Medical Science, Temerty Faculty of Medicine, University of Toronto, Toronto, ON, Canada; 2 Krembil Brain Institute, University Health Network, Toronto, ON, Canada; 3 Department of Immunology, University of Toronto, Toronto, ON, Canada; 4 Schroeder Arthritis Institute, University Health Network, Toronto, ON, Canada; 5 Department of Surgery, University of Toronto, Toronto, ON, Canada

**Keywords:** attention, chronic pain, cognitive, distraction, pain coping

## Abstract

**Introduction:**

Distraction-based coping strategies are beneficial for some people living with chronic pain. However, there is no established method to capture the individual effect of distraction from pain through task engagement that considers fluctuations in chronic pain and the clinical relevance of changes in pain. Here, we present a new scoring system that aims to capture individual differences in the effect of distraction through task engagement in the context of chronic pain and considers whether changes are clinically meaningful.

**Methods:**

We determined whether chronic pain attenuation during a task (PAT) is related to task engagement in cognitive attention-demanding numeric tasks that vary in difficulty, in people with chronic pain with different degrees of neuropathic pain based on painDETECT scores. Data from healthy controls (HCs, *n* = 27) were used to assess whether chronic pain impacted simple motor ability based on reaction time (RT) in a button-press task. In each person with chronic pain (*n* = 27), we determined their PAT scores and absolute change in chronic pain intensity and unpleasantness during both a simple motor control task and a hard task.

**Results:**

There were four main findings: (1) There was no relationship between PAT scores and task engagement in the hard task. (2) RTs were slower and more inconsistent in the chronic pain group compared to the HCs in the simple task, suggestive of diminished psychomotor abilities in the chronic pain group. (3) Mean RTs were negatively correlated with PAT scores for pain intensity in the simple task, with a greater per cent reduction of pain intensity for those with faster RTs. (4) Individuals with chronic pain who experienced increases in pain during the task had the highest painDETECT scores.

**Discussion:**

This suggests that automated tasks may be useful in temporarily lowering chronic pain intensity. Additionally, the degree of neuropathic pain may diminish the distraction effect on chronic pain. The PAT scoring system has the potential to capture the individual effect of chronic pain attenuation during task engagement, and there may be unique pain-related characteristics between PAT sub-groups that require further exploration. We encourage more validation research of PAT's utility and identify beneficiaries of attention-based pain management approaches that use distraction to manage chronic pain.

## 1 Introduction

Pain and attention share a close relationship, and each of these domains can bidirectionally influence the other process (1–3). On the one hand, pain is salient and can capture attention (2), which has evolutionarily served as a way to protect animals from bodily harm (2, 4, 5). On the other hand, allocating attention towards or away from pain can alter both nociception and the perception of pain (6). For example, seminal work in primates by Bushnell et al. (7) reported enhanced neuronal responses of wide dynamic range trigeminothalamic neurons in the medullary dorsal horn to thermal stimuli when engaged in a behaviourally relevant compared to a behaviourally irrelevant thermal task. This work demonstrated that nociception can be impacted behaviourally through attentional modulation, even at early sensory processing stages. In humans, Miron et al. (8) also showed that individuals who focused their attention on a noxious heat stimulus perceived the stimulus as more intense and unpleasant than when their attention was directed elsewhere. Furthermore, many early experimental studies have shown that attentional modulation can impact physiological and/or psychological aspects of the pain experience (9–13). The ability to alter nociception and/or aspects of pain through attention modulation has led to the development of cognitive and attention-based strategies to help cope with pain (14–20).

Distraction is one approach used as an attention-based pain-coping strategy, where attention is diverted away from an aversive experience like pain and allocated towards something else (15, 16). Many theoretical models that describe how distraction impacts pain suggest that attention is a limited cognitive resource (1, 16, 21, 22). This framework proposes that when a person engages their attention towards other demands that take up this resource (e.g., an attentionally demanding task), less attention is available to be applied towards attending to pain, thereby reducing its experience (16, 23). Intuitively, this coping strategy has its appeal because it is easy to implement in different settings (17, 24–26) and has been investigated as an attentional management approach for both acute (27–29) and chronic pain (17, 30–32). However, there are mixed results regarding the effect of distraction in significantly reducing pain (33–37), and thus whether distraction is useful as a coping strategy for chronic pain remains debatable (25). One potential contribution to the mixed results in previous studies is the heterogeneity in methodologies used to evaluate attention modulation of pain. For example, Buhle and Wager (36) address some common attention-demanding tasks used in the experimental study of task-based reduction of pain. There are many ways to develop an experimental paradigm using different attention-demanding tasks to study the attentional modulation of pain.

Several factors have hampered previous evaluations of the bidirectional pain-attention relationship. First, although the effect of attentional modulation of pain can be statistically significant, it may not be clinically meaningful. There are many ways to consider clinically meaningful changes to pain (38–40), including assessing changes to chronic pain intensity (41). In general, a ≥30% reduction in pain intensity has been considered clinically meaningful in pain and chronic pain intervention literature (38–41). Thus, a new method to evaluate distraction from pain could be useful if it were to indicate whether the effects are clinically meaningful.

Additionally, there have been reports of mixed efficacy of psychological treatments for chronic pain (42, 43). The source of these mixed results could stem from a lack of specificity in the efficacy of the components of those psychological treatments for chronic pain. For example, cognitive behavioural therapy (CBT) can involve the implementation of different coping skills, such as distraction (44). There is heterogeneity in the way CBT and its components are implemented across different clinical populations (45), and often, implementation tactics can depend on the needs of the individual. As a preliminary step towards identifying how to tailor CBT to a person's needs, it is important to first identify who could benefit from more fundamental pain-coping skills such as distraction. If the findings from such studies are framed in a way that allows for interpretation in the context of clinical intervention, they can inform the design of future studies of clinical psychological interventions for chronic pain and identify how to tailor these treatments for personalized chronic pain care (43). Second, many studies in chronic pain populations assess distraction effects on experimentally induced pain, which may not accurately model how these effects impact chronic pain (46). Third, attention towards and away from pain may wax and wane over time (47), and attentional modulation of pain could vary throughout a task. Thus, key information about the individual benefits from attentional modulation of pain could be obscured by the method and timing of pain assessment. For example, calculating average changes in pain may filter out pain ratings at time points that reached clinically relevant degrees of change. Attention towards one's own chronic pain can fluctuate, so the timing of measurement should be considered because it could determine whether someone can experience a meaningful effect from distraction. For future studies to identify people who can experience a clinically relevant analgesic effect from distraction, they should use statistical methods that consider temporal variations in changes in pain and/or the optimal timing of pain assessment. Temporal variations in pain have been considered using statistical methods to assess trial-by-trial fluctuations in the relationship between task performance and pain (36, 48–50). This approach can provide insight into the time-varying impacts of attention towards pain, but there may be other simple ways to achieve similar goals. Another less explored alternative to quickly evaluate clinically relevant changes in pain in people who experience such analgesia in only portions of a task would be to examine the point at which chronic pain attenuation is maximized for an individual during a task. Although this does not encapsulate temporal fluctuations in changes to pain, it does allow us to assess the point at which a person peaks in analgesia within a given timeframe. Thus far, there is no established approach to experimentally capture the individual effect of distraction through task engagement on a person's chronic pain that examines when chronic pain attenuation is maximized and whether these changes are clinically meaningful.

To address these limitations, we present a new scoring system to capture the individual effect of task engagement-based changes to chronic pain, which will be applied to short-duration attention-demanding tasks, and to determine whether changes in pain are clinically meaningful (i.e., ≥30% pain reduction). To do this, we characterized the maximum pain attenuation during a task (PAT) in individuals with chronic pain. Additionally, given previous studies on the impact of task demands and cognitive load on the attentional modulation of pain (35, 36, 50), we used tasks designed to vary in difficulty. Given that the PAT scoring system is exploratory, our first aim was to characterize the PAT effect in people with chronic pain across different attention-demanding tasks of varying difficulty. Our second aim was to examine whether there was a relationship between task engagement and PAT in people with chronic pain for each task. We hypothesized that people with better task performance would exhibit higher PAT than those with lower performance, and that this relationship would be more pronounced for the more difficult tasks, which were expected to require greater attentional resources.

## 2 Methods

### 2.1 Participants

All study participants provided informed consent for testing procedures that were approved by the University Health Network (UHN) Research Ethics Board. All participants underwent testing at the Toronto Western Hospital (TWH) in Toronto, Canada, as part of a larger data collection protocol for an in-house database that included neuroimaging [magnetic resonance imaging (MRI) and magnetoencephalography (MEG)], quantitative sensory testing (QST), and behavioural questionnaires. The current study only included specific components of the QST (the attention-demanding tasks) and questionnaires that were relevant to this study and were included in the analysis (see below). Healthy controls (HCs) were recruited through word of mouth and advertisements posted throughout UHN, with the inclusion criterion of being 18–65 years old and the exclusion criteria of (1) current ongoing pain or a history of chronic pain (pain that has lasted more than 3 months), (2) major chronic health condition, (3) no regular medication use (outside of birth control and vitamins), (4) a diagnosed psychiatric and/or neurological disorder, (5) a Beck Depression Inventory (BDI) (51) score higher than 13 (suggesting greater than minimal self-reported depression), and (6) no MRI/MEG contraindications. Three individuals in the HC group reported scores >13 but were retained in the study analyses because neuroimaging data were not part of this study. We also note that the reaction times (RTs) of these individuals were not slower compared to the HC group and were not outliers in our RT analyses, as could have been a confound, given that depression could contribute to slower motor performance (52). For the chronic pain group, individuals aged 18–65 years were recruited from either the Rheumatology Clinic or Pain Clinic at TWH. A total of 28 individuals were initially recruited and tested, but one was removed from analyses due to having too few trials and low per cent accuracy (below 50%) on one of the tasks (Figure 1). The participants in the chronic pain group primarily had chronic pain associated with ankylosing spondylitis (AS, a form of spondyloarthritis) alone (*n* = 17), or AS with other pain conditions (*n* = 3), while the remainder of the group had chronic pain associated with conditions other than AS (*n* = 7) (e.g., disc degeneration, sciatica, migraines) (Supplementary Figure S1). We included individuals who self-reported low or inconsistent pain (i.e., average pain that was under 3/10 on the numeric rating scale for pain intensity and pain that was managed well with medications), given the exploratory nature of the study to assess the impact of distraction across pain intensities.

**Figure 1:**
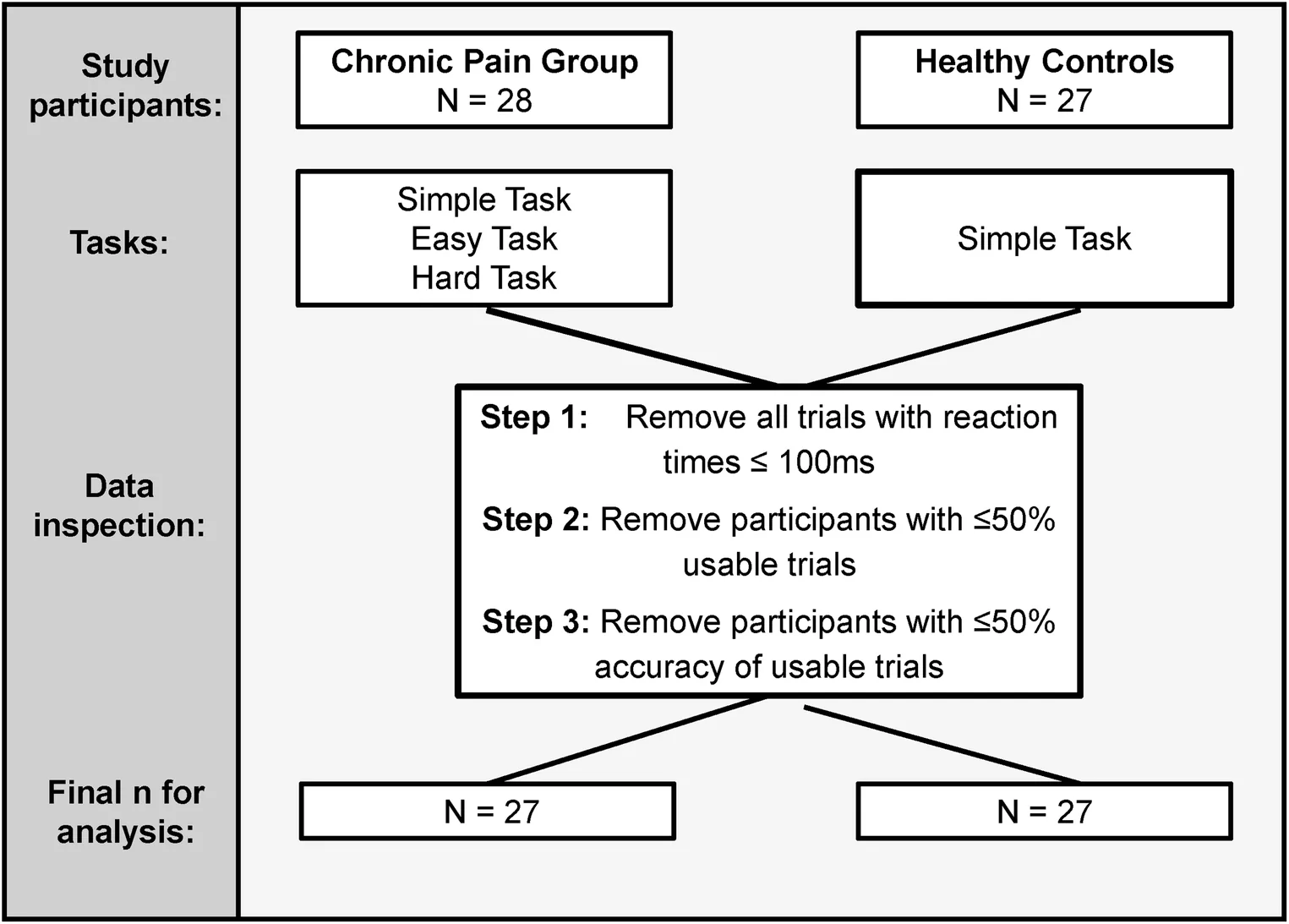
Study participants and workflow overview. Overview of the number of study participants in each cohort (healthy controls and chronic pain group) and the tasks each group underwent. The criteria used during the data inspection phase to remove trials and participants are also shown.

### 2.2 Attention-demanding tasks

An overview of the tasks and experimental design is shown in Figure 2. All tasks were run on E-Prime 3.0 (3.0.3.214) Psychological Software Tools. We used three levels of task difficulty to examine cognitive/attentional demands and motor abilities (Figure 2A). A simple motor task was used to evaluate fundamental motor and cognitive engagement/abilities in both the HC and chronic pain groups. Additionally, individuals in the chronic pain group were tested at two additional levels of task difficulty (easy, hard), which were shorter versions of the numerical interference task we have used previously (53). For all tasks, participants viewed a computer screen that displayed rectangles containing numbers (i.e., numeric “cards”). The duration of each task (excluding instructions and practice trials) was approximately 6 min.

**Figure 2:**
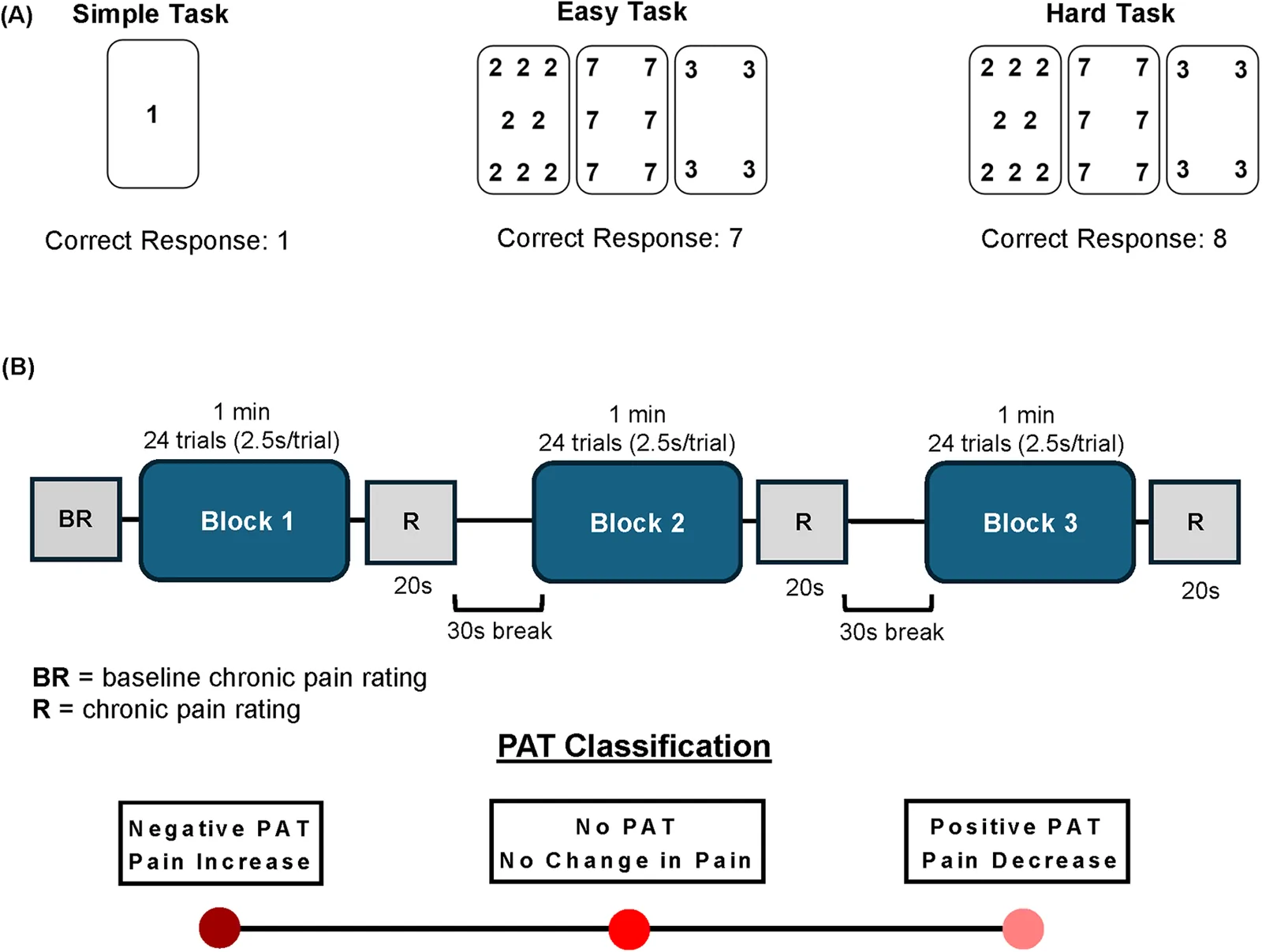
Attention-demanding tasks and experimental design. **(A)** Examples of the visual display for each of the three attention-demanding tasks (simple motor control, easy and hard). **(B)** Overview of the experimental design, which consisted of three blocks of 24 task trials preceded and followed by obtaining a rating of chronic pain. The pain attenuation during task (PAT) classification designation to categorize the participant as having either negative, no, or a positive PAT is also shown.

In the simple task, a single card was displayed that contained one number (1, 2, or 3 presented in ascending order sequentially in each trial), and participants were instructed to indicate which number was displayed on the card by pressing the corresponding number on a keypad as quickly and accurately as possible. This was essentially a tapping motor task.

In the easy and hard tasks, three rectangular cards were displayed side by side. Each card contained several repetitions of single digits (i.e., from 1–9). For example, a card may have eight repetitions of the number two. Each card had a unique digit and repetition of that digit. In the easy task, participants were asked to examine the values of the digits represented on the three cards and report the largest value by pressing the corresponding button on the number keypad as quickly and accurately as possible. For the hard task, participants were asked to compare the number of digits represented on the cards and report the largest number of digits by pressing the corresponding button on the number keypad as quickly and accurately as possible. All tasks consisted of three 1-minute blocks, each of which contained twenty-four 2.5-s trials. People with chronic pain were asked to rate their pain intensity from 0 to 100 (0—no pain, 100—worst pain imaginable) and unpleasantness from 0 to 100 (0—not unpleasant at all, 100—most unpleasant imaginable) just prior to the first block, and after each block (just before the inter-block interval) (Figure 2B). For the pain ratings prior to the tasks, participants were instructed to rate the current chronic pain (i.e., what is the intensity of your chronic pain NOW on a scale from 0 to 100). For the pain ratings after each block, participants were instructed to rate their chronic pain levels they experienced during the block (i.e., what was the intensity of your chronic pain DURING the block on a scale from 0 to 100). Before the tasks, participants were given a card example, timed practice trials, and an opportunity to practice rating their chronic pain using the numeric keypad. For the chronic pain group, the tasks were presented in ascending order of difficulty.

### 2.3 Data checks and task engagement metrics

Task engagement was quantified for each participant based on metrics of their task performance: mean reaction time (RT), standard deviation (SD), and per cent accuracy (% Acc) in each task for the trials that passed all data checks. Both correct and incorrect trials were used for analyses. The % Acc represented the percentage of usable trials with correct responses. The data checks used to remove inappropriate trials were as follows (Figure 1):

In step 1, trials with RTs ≤ 100 ms were removed. This cut-off was based on the postulated minimum time needed for stimulus detection, decision-making, and a motor response (54). In step 2, participants were removed from analyses if ≤50% of their trials remained after step 1. In step 3, participants with % Acc of ≤50% within usable trials were removed from the analyses. This last step aimed to remove participants who performed at chance level or below in performance and could potentially be guessing rather than actively engaging with the tasks. One participant was removed because they had too few trials and low per cent accuracy on the hard task. For the HCs in the simple motor task, 18 people had 0 trials removed, five people had one trial removed, two people had two trials removed, one person had three trials removed, and one person had 16 trials removed. In the same task for the chronic pain group, 24 people had 0 trials removed, two people had one trial removed, and one person had two trials removed. In the easy task, 17 people had 0 trials removed, four people had one trial removed, one person had two trials removed, three people had three trials removed, one person had four trials removed, and one person had seven trials removed. In the hard task, eight people had 0 trials removed, five people had one trial removed, three people had two trials removed, two people had three trials removed, three people had four trials removed, two people had five trials removed, one person had six trials removed, one person had seven trials removed, one person had eight trials removed, and one person had 19 trials removed.

### 2.4 Change in pain characterization: pain attenuation during a task (PAT) and absolute change in pain

The effect of task engagement on chronic pain was quantified for each person in each task using two metrics: (1) PAT scores and (2) absolute change in pain. Both measurements reflect changes in pain that represent the maximum pain attenuation in an individual during a task. In total, there were four outcomes of maximum pain attenuation: pain intensity (% change, PAT), pain unpleasantness (% change, PAT), absolute change in pain intensity, and absolute change in pain unpleasantness. PAT scores are calculated as a per cent change from baseline state pain rating (pain rating provided just before the task starts) to the post-block retrospective rating in the task block with the greatest pain attenuation. If a participant did not exhibit a pain decrease, then the task block with the retrospective pain rating closest to zero was used to generate their score. Positive PAT scores represent a decrease in pain during the task, scores of zero indicate no change in pain during the task, and negative scores indicate an increase in pain during the task (Figure 2B). PAT scores could not be determined for some individuals who did not have any pain prior to the tasks tested (baseline state pain rating = 0). To assess PAT in these individuals, we calculated their absolute change in pain from baseline to the block with the post-block pain rating closest to zero for each participant. This was done to avoid a zero-division issue in the PAT score calculation. The equations for PAT scores and absolute change in pain are given below:

1. $\mathrm{PAT}\;\mathrm{Score}=\;\left(\frac{\mathrm{Baseline}\;\mathrm{State}\;\mathrm{Pain}\;\mathrm{Rating}\text{-}\mathrm{Post}\;\mathrm{Task}\;\mathrm{Block}\;\mathrm{Pain}\;\mathrm{Rating}}{\mathrm{Baseline}\;\mathrm{State}\;\mathrm{Pain}\;\mathrm{Rating}}\right)\;\times 100$
2. AbsoluteChangeinPain=(BaselineStatePainRating-PostTaskBlockPainRating)

### 2.5 Analyses

Analyses of normality, skewness, and kurtosis were used to assess the distribution of the data and indicated that non-parametric statistical tests should be used for the study analyses. Statistical analyses and figures were generated using GraphPad Prism (version 7.03, https://www.graphpad.com). Effect size calculations were made using R-Studio (version 2026.01.2 + 418), with the libraries “effectsize” (for rank biserial correlation), “DescTools” (for Kendall’s *W* test), and “rstatix” (for ordinal eta squared test). The standard significance threshold before multiple comparisons was set to *α* = 0.05.

#### 2.5.1 Task engagement between HC and chronic pain group on simple task

Baseline motor response and task engagement were compared between the HCs and chronic pain group in the simple task to identify normative performance in RTs and to assess if there was diminished motor ability in the chronic pain group. Mean RT and RT SD were calculated using all usable trials cumulatively across the three blocks for every participant. Mann–Whitney *U*-tests were used to examine group-level differences in mean RTs and RT SD, respectively, between the HCs and the chronic pain group. We used non-parametric tests to report the group medians of the mean RT and RT SD in both groups, respectively. This analysis was not applied to the % Acc data because a preliminary visualization showed a ceiling effect in performance for both groups. Effect sizes were reported using rank biserial correlations (r_rb_).

#### 2.5.2 Assessing task difficulty

To examine the effect of task difficulty on the relationship between task engagement and measurements of change in pain (PAT and absolute change in pain) in subsequent analyses, we first checked that the study tasks varied in difficulty at both the subject and group levels. Thus, we calculated the within-subject differences in task engagement metrics (RT, RT SD, and % Acc) and used non-parametric Friedman's tests with Dunn's test for *post-hoc* pairwise comparisons between all three tasks for each task engagement metric, respectively, to determine whether participants exhibited better performance on the easier tasks compared to the harder tasks (i.e., faster RTs, less variability in RTs, and a higher percentage of correct trials). Tasks were considered different in difficulty if they showed a significant difference in all three task engagement metrics, with lower performance as tasks ascended in difficulty. Bonferroni corrections were used for multiple comparisons. Mean RT, RT SD, and % Acc were calculated using all usable trials cumulatively across the three blocks in each task separately for every participant. Since we used a non-parametric test, group medians for mean RT, RT SD, and % Acc were reported for each task. Effect sizes were reported using Kendall's *W* tests (*W*).

#### 2.5.3 Assessing the relationship between the pain metrics and task engagement

The relationships between pain metrics (PAT and absolute change in pain) and task engagement metrics (mean RT, RT SD, and % Acc) generated from all usable trials cumulatively in all three blocks were assessed for each task separately using Spearman's correlations. Bonferroni corrections were used to correct for multiple comparisons.

#### 2.5.4 Additional pain-related assessments (supplemental exploratory analysis)

An additional Kruskal–Wallis test was used to compare group-level differences in pain-related assessments between PAT sub-groups (positive PAT, no PAT, and negative PAT). The pain-related assessments included the Brief Pain Inventory (55–57) (BPI) (pain severity: 0—no pain, 10—worst pain; pain interference: 0—does not interfere, 10—completely interferes), the painDETECT (58) [0–38; ≤12: low likelihood of neuropathic pain (NP), 13–18: unclear if NP is present, ≥19: high likelihood of NP], baseline trait pain (taken from the painDETECT), and baseline state pain intensity just prior to each task (0–100; 0—no pain, 100—worst pain imaginable). The baseline trait pain ratings were extracted from the question: how strong was the pain during the last 4 weeks on average? The responses can range from 0 (none) to 10 (maximum). Since we used a non-parametric test, group medians of each metric were reported for each sub-group. Effect sizes were reported using ordinal eta squared (*η*^2^_H_).

## 3 Results

### 3.1 Study participants and PAT distribution

In total, after removing one participant during the data inspection phase, there were 27 HCs [13 males, 14 females; mean ± standard deviation (SD) age = 26.9 ± 6.6 years] and 27 people with chronic pain (14 males, 13 females; mean ± SD age = 38.6 ± 9.3) used for the study analyses (Figure 1). A Mann–Whitney *U*-test to assess differences in group ages revealed that the chronic pain group was significantly older than the healthy individuals (*U* = 119, *p* < 0.0001). Based on the PAT scores for pain intensity from the simple task, six people experienced a decrease in pain, 12 people had no changes to their pain, and four people experienced an increase in pain. Of the six people with a decrease in pain, five experienced clinically relevant changes to their pain intensity. For the PAT scores regarding pain unpleasantness in the simple task, six people experienced a decrease in pain, 13 people experienced no changes to their pain, and 1 person experienced an increase in pain (Table 1). Based on the PAT scores for pain intensity for the hard task, four people experienced a decrease in pain, 13 people had no changes to their pain, and four people experienced an increase in pain. There were no people with clinically relevant changes to their pain intensity. For the PAT scores regarding pain unpleasantness in the hard task, five people experienced a decrease in pain, 11 people experienced no changes to their pain, and five people experienced an increase in pain (Table 2). For the distribution of individuals by the absolute pain ratings, see Tables 1, 2.

**Table 1:** PAT distribution in the simple task.

Number of participants in pain groups simple task				
Variables	Pain decrease	No change to pain	Pain increase	≥30% Pain intensity decrease
Pain intensity (% change, PAT)	6	12	4	5
Pain unpleasantness (% change, PAT)	6	13	1	NA
Pain intensity (absolute change)	6	16	5	NA
Pain unpleasantness (absolute change)	6	18	2	NA

The number of individuals in each pain attenuation during task (PAT) group and absolute change in pain for intensity and unpleasantness for the simple task are shown in the table. Also shown is the number of individuals who have experienced a clinically relevant decrease in pain intensity. NA, not applicable.

**Table 2:** PAT distribution in the hard task.

Number of participants in pain groups hard task				
Variables	Pain decrease	No change to pain	Pain increase	≥ 30% Pain intensity decrease
Pain intensity (% change, PAT)	4	13	4	0
Pain unpleasantness (% change, PAT)	5	11	5	NA
Pain intensity (absolute change)	4	19	4	NA
Pain unpleasantness (absolute change)	5	17	5	NA

The number of individuals in each pain attenuation during task (PAT) group and absolute change in pain for intensity and unpleasantness for the hard task are shown in the table. Also shown is the number of individuals who have experienced a clinically relevant decrease in pain intensity. NA, not applicable.

### 3.2 Task engagement in the simple task between healthy controls and people with chronic pain

To assess whether there may be diminished motor ability in the chronic pain group, between-group differences in normative reaction time performance were assessed for the simple task. The mean RTs and variability in RTs are shown in Figure 3. The chronic pain group (median = 421 ms) exhibited significantly slower RTs compared to the HC group (median = 328 ms) (U = 174, *p* = 0.0008, *r*_rb_ = −0.52) (Figure 3). The chronic pain group (median = 172 ms) also exhibited significantly greater variability in RTs compared to the HC group (median = 88 ms) (*U* = 174, *p* = 0.0008, *r*_rb_ = −0.52) (Figure 3).

**Figure 3:**
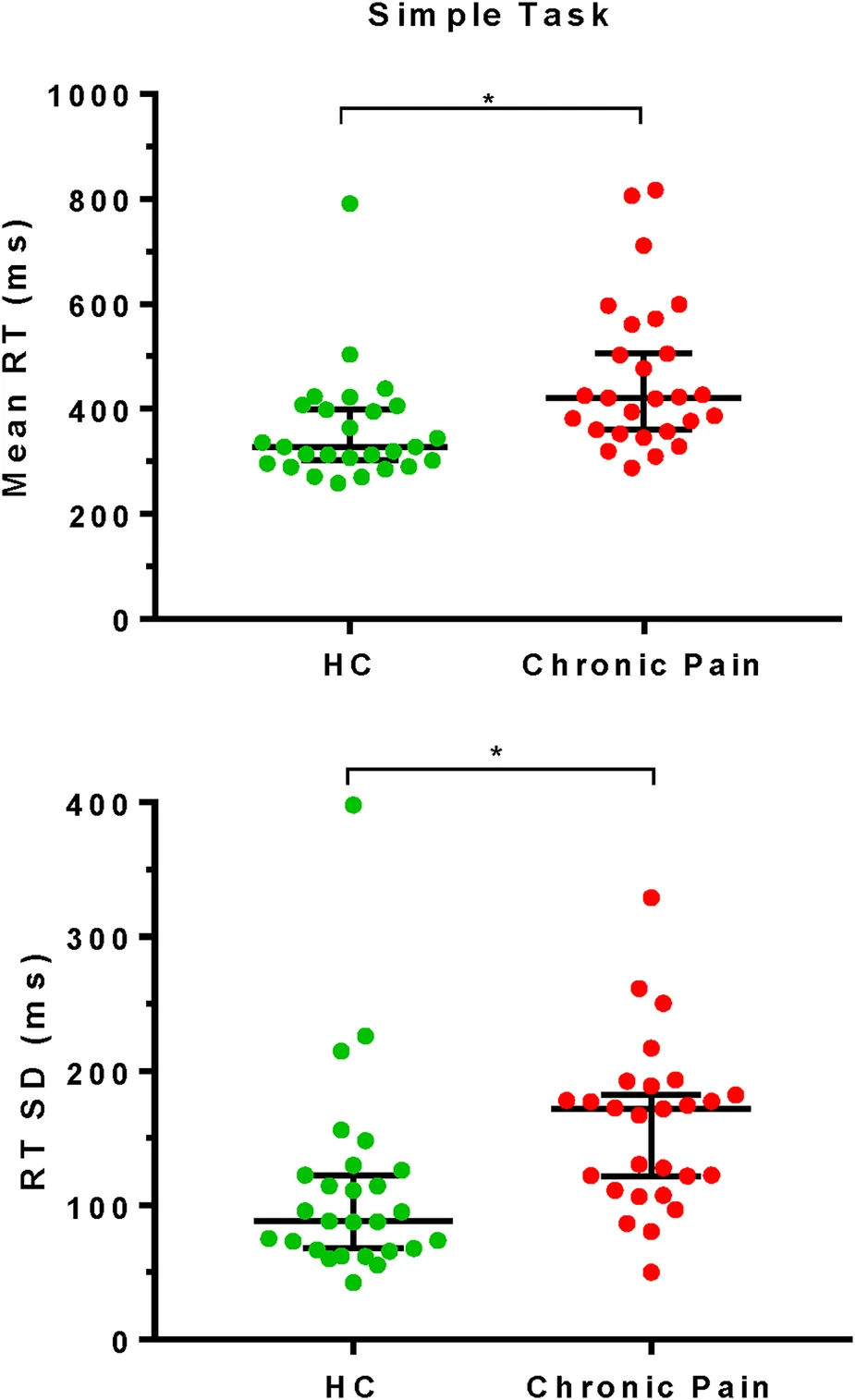
Simple motor control task reaction times in the healthy control and chronic pain groups. The individual and group reaction times (RT) and variability [RT standard deviation (SD)] are shown for the 27 healthy controls (HC, green circles) and 27 people with chronic pain (red circles) for the simple motor control task. Significant differences were observed in mean RT and RT SD, respectively, such that the chronic pain group had overall slower and more inconsistent RTs compared to the HCs (*p* = 0.0008). The * symbol represents significant findings where *p*-values are ≤0.05. Horizontal lines in the data represent the group median and 95% confidence intervals.

### 3.3 Task difficulty assessment

The performance metrics were used to assess whether the different tasks varied in difficulty. A within-group comparison of task difficulty across the three tasks was conducted for the chronic pain group based on the performance metrics of mean RT, variability in RT, and % Acc (see Figure 4). For the first indicator of task difficulty, RT, at the individual level, the mean RTs were significantly different between the simple (median = 421 ms), easy (median = 1278 ms), and hard tasks (median = 1,443 ms) (Friedman's statistic = 47.19, *p* < 0.0001, *W* = 0.70)*.* The pairwise statistical differences were found between the simple vs. easy (*p*_adj < 0.0001), simple vs. hard (*p*_adj < 0.0001), and easy vs. hard (*p*_adj = 0.029) tasks. Thus, overall, the RTs were slower in the hard task compared to the other tasks (Figure 4).

**Figure 4:**
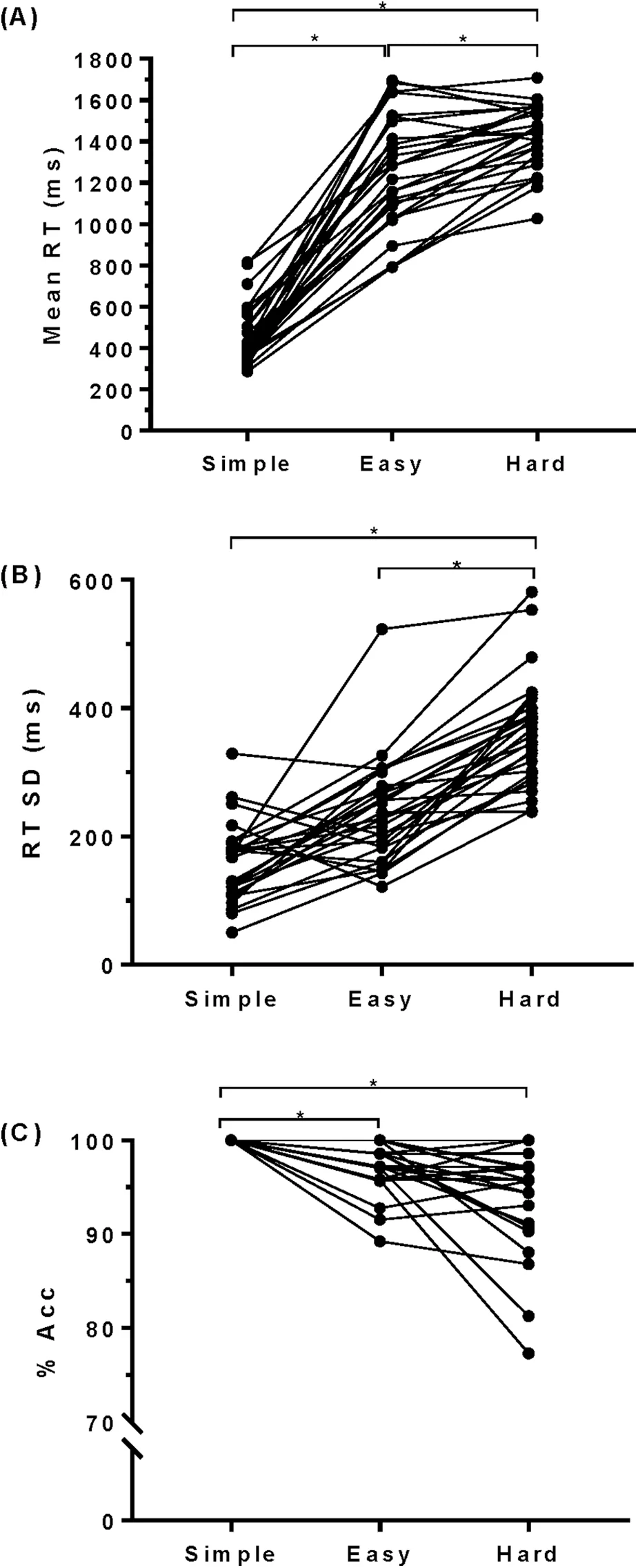
Task speed, variability, and per cent accuracy across tasks. The individual **(A)** reaction times (RT), **(B)** variability [RT standard deviation (SD)], and **(C)** per cent accuracy (% Acc) are shown for the 27 people with chronic pain for the simple motor control, easy, and hard tasks. Significant differences in RT were found between simple vs. easy (*p* < 0.0001), simple vs. hard (*p* < 0.0001), and easy vs. hard tasks (*p* = 0.029). Significant differences were noted in RT variability between simple vs. hard (*p* < 0.0001) and easy vs. hard (*p* < 0.0001), but not between the simple vs. easy task (*p* = 0.12). Significant differences were noted in % Acc between simple vs. easy (*p* = 0.0002) and simple vs. hard (*p* < 0.0001), but not between the easy vs. hard task (*p* = 0.74). The * symbol represents significant findings where *p*-values are ≤0.05.

For the second indicator of task difficulty, there were significant differences in the variability of RTs (i.e., RT SD) between the simple (median = 172 ms), easy (median = 228 ms), and hard tasks (median = 357 ms) (Friedman's statistic = 44.67, *p* < 0.0001, *W* = 0.55). The main pairwise statistical differences were found between the hard vs. simple (*p*_adj < 0.0001) and easy (*p*_adj < 0.0001) tasks, but there were no statistically significant differences between the simple vs. easy task (*p*_adj = 0.12). Furthermore, for the significant pairs, RTs were less variable in the simple and easy tasks than in the more difficult task (Figure 4).

For the third indicator of task difficulty assessed, there was a significant difference in % Acc between the simple (median = 100%), easy (median = 97.2%), and hard tasks (median = 95.7%) (Friedman's statistic = 33.15, *p* < 0.0001, *W* = 0.38). The pairwise statistical differences were found between the simple vs. easy task (*p*_adj = 0.0002) and hard task (*p*_adj < 0.0001), but not between the easy vs. hard tasks (*p*_adj = 0.74). For the significant pairs, % Acc was higher in the easier tasks compared to the harder tasks (Figure 4).

Thus, the assessment of task difficulty revealed that the easy and hard tasks did not show robust differences, and therefore we streamlined the analyses to examine the relationship between task engagement and change in chronic pain (PAT and absolute change in pain) to focus on the simple and hard tasks. For more information on within-subject performance across the blocks of our tasks, please see Supplementary Figures S2-S5.

### 3.4 Relationship between task engagement and change in pain intensity and unpleasantness

To assess whether the PAT scoring system captured a distraction effect and did not just reflect within-subject temporal fluctuations of chronic pain, we tested whether greater task engagement was related to greater pain reduction in each task separately. To do this, we examined the relationships between the task engagement metrics (mean RT, RT SD, and % Acc) and the measurements of maximum change in pain [pain intensity (% change, PAT), pain unpleasantness (% change, PAT), absolute change in pain intensity, and absolute change in pain unpleasantness] during a task. There was a significant negative correlation between pain intensity (% change, PAT) and mean RTs in the simple task (rho = −0.57, *p* = 0.0053), such that greater per cent reductions in pain intensity were associated with faster RTs (Figure 5A). This finding also passed Bonferroni correction for multiple comparisons. Additionally, there was a negative relationship trending towards significance between absolute change in pain intensity and mean RTs (rho = −0.35, *p* = 0.071) but was not significant after Bonferroni correction for multiple comparisons (Figure 5A). There was no significant relationship between pain intensity (% change, PAT) and mean RT (rho = −0.22, *p* = 0.33) in the hard task (Figure 5B). There was also no significant relationship between absolute change in pain intensity and mean RT (rho = −0.18, *p* = 0.37) in the hard task (Figure 5B). There were no other significant correlations between the remaining measurements for maximum change in pain and task engagement for the simple and hard tasks, respectively. For a summary of the outcomes of these correlations, see Tables 3, 4.

**Figure 5:**
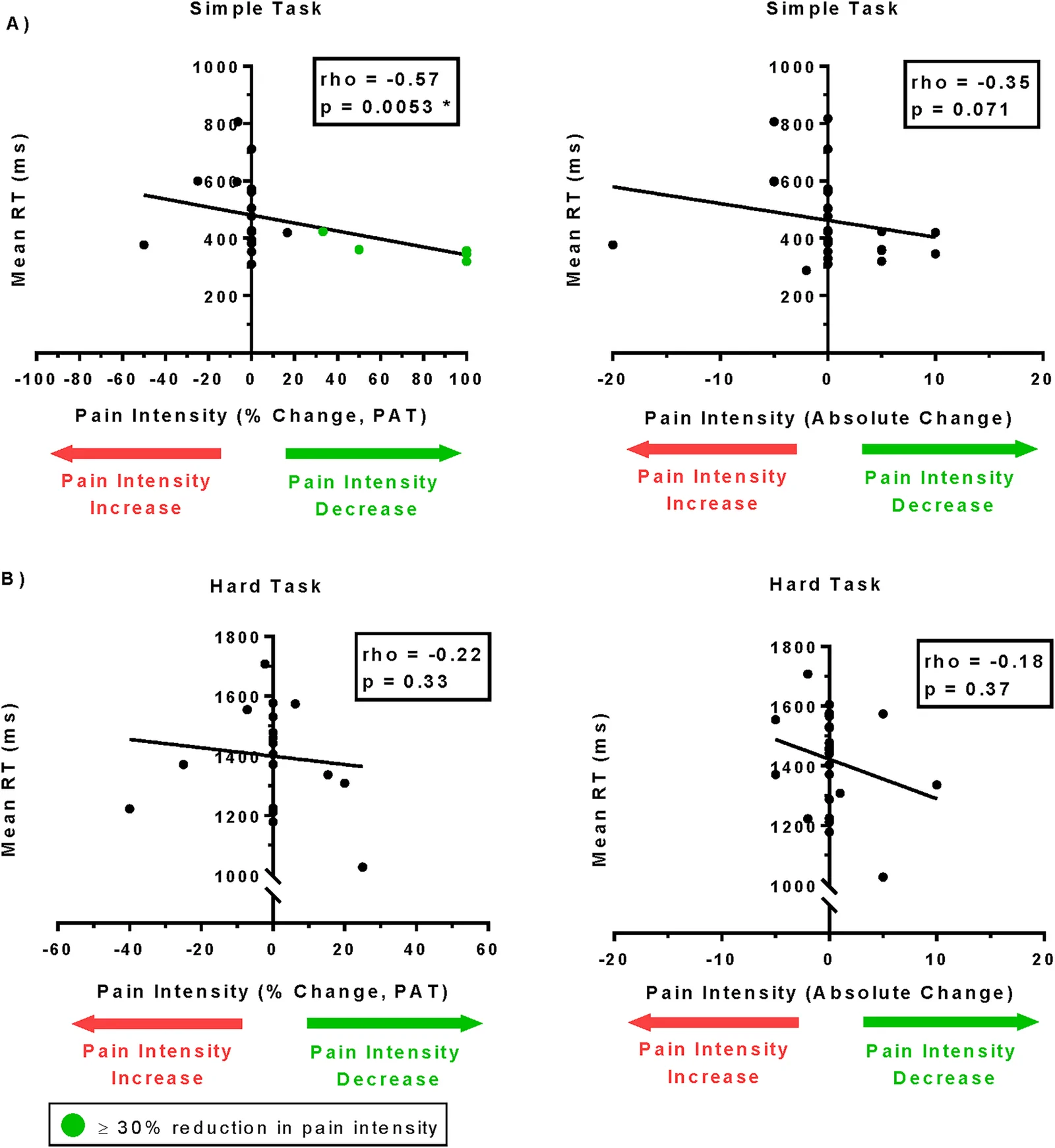
Correlation between reaction time and pain intensity in the simple task. The relationship between mean reaction time (RT) vs. pain attenuation during task (PAT) score for pain intensity and absolute change in pain intensity is shown for the chronic pain group. **(A)** In the simple task, faster RTs are significantly associated with a higher per cent pain decrease (*p* = 0.0053) and trending towards a significant association with a higher absolute change in pain intensity (*p* = 0.071). **(B)** In the hard task, there were no significant correlations between RTs and per cent change in pain intensity (*p* = 0.33) or absolute change in pain intensity (*p* = 0.37). The * symbol represents significant findings where *p*-values are ≤ 0.05.

**Table 3:** Spearman's correlations between task engagement and pain metrics for the simple task.

Spearman's correlations simple task				
Variables	(E) Mean RT	(F) RT SD	(G) % Acc	Significance
(A) Pain intensity (% change, PAT)	rho = −0.57 *p* = 0.0053	rho = −0.33 *p* = 0.13	NA	A vs. E* A vs. F, n.s
(B) Pain unpleasantness (% change, PAT)	rho = - 0.14 *p* = 0.56	rho = −0.14 *p* = 0.57	NA	B vs. E, n.s B vs. F, n.s
(C) Pain intensity (absolute change)	rho = −0.35 *p* = 0.071	rho = −0.14 *p* = 0.49	NA	C vs. E, n.s C vs. F, n.s
(D) Pain unpleasantness (absolute change)	rho = 0.062 *p* = 0.76	rho = 0.12 *p* = 0.55	NA	D vs. E, n.s D vs. F, n.s

The results for Spearman's correlations between the task engagement metrics [mean reaction time (RT), RT standard deviation (SD), and per cent accuracy (%Acc)] and the pain attenuation during task (PAT) metrics in the simple task are shown in the table. NA, not applicable; n.s, not significant.

The * symbol represents significant findings where *p*-values are ≤0.05.

**Table 4:** Spearman's correlations between task engagement and pain metrics for the hard task.

Spearman's correlations hard task				
Variables	(E) Mean RT	(F) RT SD	(G) % Acc	Significance
(A) Pain intensity (% change, PAT)	rho = −0.22 *p* = 0.33	rho = −0.060 *p* = 0.80	rho = −0.13 *p* = 0.57	A vs. E, n.s A vs. F, n.s A vs. G, n.s
(B) Pain unpleasantness (% change, PAT)	rho = −0.24 *p* = 0.30	rho = −0.28 *p* = 0.23	rho = −0.10 *p* = 0.66	B vs. E, n.s B vs. F, n.s B vs. G, n.s
(C) Pain intensity (absolute change)	rho = −0.18 *p* = 0.37	rho = −0.028 *p* = 0.89	rho = −0.11 *p* = 0.60	C vs. E, n.s C vs. F, n.s C vs. G, n.s
(D) Pain unpleasantness (absolute change)	rho = −0.18 *p* = 0.38	rho = −0.17 *p* = 0.39	rho = −0.11 *p* = 0.57	D vs. E, n.s D vs. F, n.s D vs. G, n.s

The results for Spearman's correlations between the task engagement metrics [mean reaction time (RT), RT standard deviation (SD), and per cent accuracy (%Acc)] and the pain attenuation during task (PAT) metrics in the hard task are shown in the table. NA, not applicable; n.s, not significant.

### 3.5 Pain-related assessments between PAT sub-groups

We ran an additional analysis to determine whether there were between-group differences in each of the pain-related assessments (see Methods section 2.5.4) in the PAT sub-groups during the simple task, which was the only task that exhibited a significant relationship between PAT for pain intensity and mean RT. We found that the painDETECT scores exhibited significant between-group differences for the positive PAT (median = 3.5), no PAT (median = 7.5), and negative PAT groups (median = 16) (*H* = 6.83, *p* = 0.025, *η*^2^_H_ = 0.25). The pairwise statistical tests identified a difference between the positive PAT and negative PAT groups (*p*_adj = 0.029), but not between the positive PAT vs. no PAT groups (*p*_adj = 0.87) or the negative PAT vs. no PAT groups (*p*_adj = 0.14). Those who experienced chronic pain attenuation during the simple task (positive PAT group) had overall lower painDETECT scores (i.e., less likelihood of NP) than those who experienced chronic pain increase (negative PAT group) (Figure 6A).

**Figure 6:**
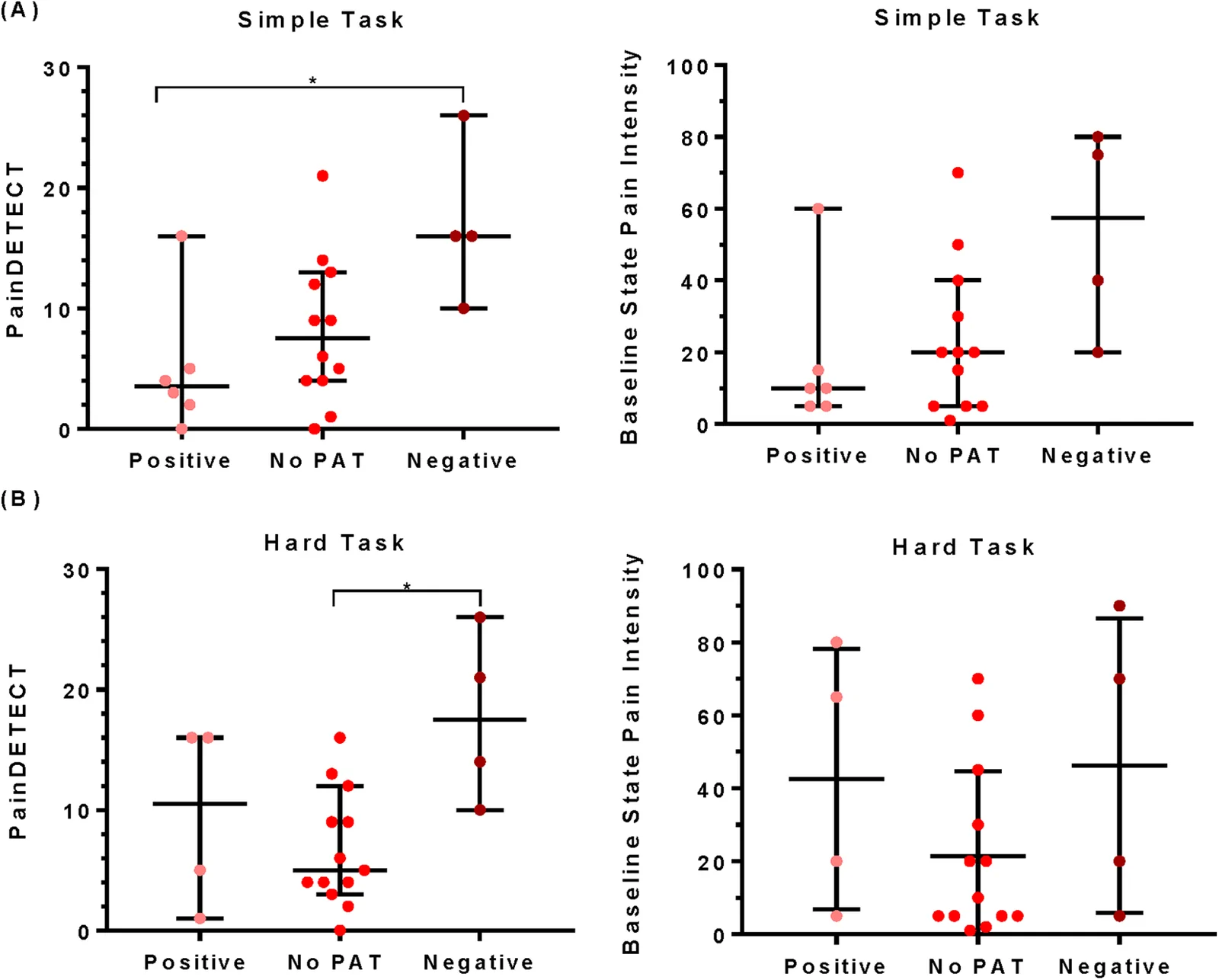
PainDETECT scores and baseline state pain intensity ratings. The individual and group painDETECT scores and baseline state pain intensity ratings are shown for the pain attenuation during task (PAT) sub-groups (positive PAT, light red circles; no PAT, red circles; negative PAT, dark red circles). **(A)** In the simple task, significant differences in painDETECT scores were observed between the positive PAT vs. negative PAT groups (*p* = 0.029), but not between the positive PAT vs. no PAT groups (*p* = 0.87) or the negative PAT vs. no PAT groups (*p* = 0.14). There was a trend towards significant differences in baseline state pain intensity ratings between the positive PAT vs. negative PAT groups (*p* = 0.07), but not between the positive PAT vs. no PAT groups (*p* > 0.99) or the negative PAT vs. no PAT group (*p* = 0.16). **(B)** In the hard task, significant differences in painDETECT scores were between no PAT vs. negative PAT groups (*p* = 0.042), but not between the positive PAT vs. no PAT groups (*p* = >0.99) or the negative PAT vs. positive PAT groups (*p* = 0.54). There were no significant differences between PAT groups in baseline state pain intensity ratings (*p* = 0.26). The * symbol represents significant findings where *p*-values are ≤0.05. Horizontal lines in the data represent the group median and 95% confidence intervals.

Additionally, there was a trend towards a significant difference in baseline state pain intensity between the positive PAT (median = 10), no PAT (median = 20), and negative PAT groups (median = 57.5) (*H* = 5.46, *p* = 0.059, *η*^2^_H_ = 0.18). The pairwise difference trending towards significance was between the positive and negative PAT groups (*p*_adj = 0.07), but not between the positive PAT vs. no PAT group (*p*_adj > 0.99) or the negative PAT vs. no PAT group (*p*_adj = 0.16). The positive PAT group had an overall lower pain intensity at the start of the simple task compared to the negative PAT group (Figure 6A). There were no other significant differences between the remaining pain-related assessments (see Table 5).

**Table 5:** Pain-related measurements in PAT groups of simple task.

Pain-related measurements in PAT groups of simple task				
Variables	Positive PAT (*n* = 6)	Zero PAT (*n* = 12)	Negative PAT (*n* = 4)	Significance (*p*-value), (*η*^2^_H_)
*N* [M (%), F (%)]	[4 (67%), 2 (33%)]	[5 (42%), 7 (58%)]	[1 (25%), 3 (75%)]	NA
Age (years)	38.5 ± 11.7	38.2 ± 7.6	39.8 ± 11.6	NA
Baseline state pain intensity (0–100)	17.5 ± 19.3	23.4 ± 20.0	53.8 ± 24.8	0.059, 0.18
Baseline trait pain intensity (0–10)	3.5 ± 2.9	4.4 ± 1.9	6.0 ± 1.9	0.24, 0.05
PainDETECT (0–38)	5.0 ± 5.2	8.2 ± 5.8	17 ± 5.8	0.025*, 0.25
BPI severity (0–10)	2.8 ± 2.4	3.5 ± 2.1	5.0 ± 1.9	0.29, 0.029
BPI interference (0–10)	2.5 ± 2.8	3.5 ± 2.4	4.9 ± 3.2	0.34, 0.014

The average group scores (mean ± standard deviation) for each PAT group for pain intensity in the simple task are shown in the table. Also shown are the results of the Kruskal–Wallis tests that examine group-level differences in the metrics between groups. The significance column has the *p*-value and the ordinal eta squared (*η*^2^_H_) effect size values. NA, not applicable.

The * symbol represents significant findings where *p*-values are ≤0.05*.*

We assessed between-group differences in painDETECT scores and baseline state pain intensity between the PAT sub-groups of the hard task. We found that the painDETECT scores exhibited significant between-group differences for the positive PAT (median = 10.5), no PAT (median = 5), and negative PAT groups (median = 17.5) (*H* = 6.09, *p* = 0.039, *η*^2^_H_ = 0.23). The pairwise statistical tests identified a difference between the no PAT and negative PAT groups (*p*_adj = 0.042), but not between the positive PAT vs. no PAT group (*p* = >0.99) or the negative PAT vs. positive PAT group (*p* = 0.54). Those who experienced no changes to their chronic pain during the hard task (no PAT group) had overall lower painDETECT scores (i.e., less likelihood of NP) than those who experienced an increase in chronic pain (negative PAT group) (Figure 6B). There were no significant differences in baseline state pain intensity between the PAT sub-groups of the hard task (*H* = 2.76, *p* = 0.26, *η*^2^_H_ = 0.042) (Figure 6B). For more information on trait and state chronic pain of our participants during our tasks, please see Supplementary Figure S6.

## 4 Discussion

The interaction between pain and attention has been shown to bidirectionally influence one another (1–3), but it has been largely studied in the context of experimental pain, with less known about the effects related to chronic pain itself. For example, experimentally induced pain can impact task performance on attention-demanding cognitive tasks in healthy people (53, 59–62), but it is challenging to disentangle whether the impacts on performance in cognitive tasks arise from the influence of experimentally induced pain or chronic pain (46). Reciprocally, distraction has been shown to be a behavioural coping strategy for acute and clinical pain that can produce modest pain-attenuating effects (36, 48–50, 63–69), but its effectiveness on chronic pain is inconclusive. First, there is the issue of individual variability in the chronic pain experience over time, as chronic pain and attention can fluctuate, waxing and waning (47). Second, there is the issue of the effect of distraction at the individual level on attributes of their chronic pain (e.g., pain type, pain intensity, pain unpleasantness, pain interference with activities of daily living). Third, there is the issue of whether the effects of distraction on chronic pain rise to the level of clinical relevance (25, 30, 31). The current study was designed to address these gaps in previous studies of chronic pain-attention interactions. Thus, the new scoring system presented here was designed to evaluate an individual's maximum pain attenuation from task engagement, which can assess the clinical relevance of the distraction effect (e.g., if there is a ≥30% reduction in pain intensity). We applied this system to attention-demanding tasks that were of short duration. Our main findings were that people with chronic pain: (1) had slower and more variable RTs in a simple motor task compared to HCs, (2) experienced greater attenuation of their chronic pain intensity if they had faster RTs in the simple task, and 3) generally, those with more neuropathic components experienced an increase in chronic pain during task engagement. These findings suggest that the PAT scoring system can potentially capture the distraction effect of task engagement on chronic pain. Furthermore, the findings highlight preliminary evidence of potential differences in the degree of neuropathic pain in those who experienced increased chronic pain during the tasks compared to those who experienced no changes or a distraction effect.

The finding of longer RTs in the chronic pain group compared to HCs on the simple motor task suggests an overall motor or psychomotor slowing in the chronic pain group. This finding is important as it indicates that task engagement (specifically in the RT-related measurements) was likely impacted by motor slowing in people with chronic pain. Additionally, the impact of chronic pain on cognitive performance found in the current study aligns with our previous studies that reported “sluggish” RTs on a Stroop Task in people with temporomandibular disorder (70) and inconsistent RTs on the numeric interference task in people with AS (46) compared to healthy controls. Our findings also contribute to the growing evidence of diminished cognitive processes such as attention, memory, information processing, and psychomotor function in people with chronic pain (71–76).

In the current study, disease symptomatology and associated comorbidities of the chronic pain group could have impacted their motor performance on the simple task. It is notable that many individuals in the chronic pain group likely had neuropathic pain based on their painDETECT scores. Neuropathic pain arises from lesions or diseases of the somatosensory nervous system (77). Those with neuropathic pain can present with sensations that are attention-demanding, such as burning, prickling, electric shock-like, spontaneous, and paroxysmal pain (77). Additionally, some individuals in the chronic pain group had pain from conditions that can also affect the upper extremities (see Supplementary Figure S1). A disruptive effect of neuropathic pain symptoms and pain in locations that can impact other aspects of motor performance could impact the consistency and speed of executing motor responses. As such, disease symptomatology and pain location may have played a role in the slower and inconsistent reaction times in the chronic pain group for the simple motor task. However, a limitation of our study is that the relatively small sample size precluded statistical analyses to examine whether motor performance was impacted by pain type or pain location. Furthermore, due to similar sample size issues, we could not assess whether clinical factors such as disease severity, fatigue at the time of testing, or medication effects impacted motor abilities.

We did find significant differences in ages between our HCs and chronic pain group. The HCs were significantly younger, which may have contributed to the faster and less variable RTs in this population. Other studies that have examined RTs in simple motor tasks show that RTs increase with age (78, 79). However, longitudinal studies have demonstrated that accelerated changes in simple RTs generally occur closer to 50 years of age and above (79). A study by Wilkinson and Allison (80) found that group averages in RTs and RT variability on a simple motor task were not significantly different between individuals in their 20s and 30s. Nevertheless, given that RTs do show an incremental increase with age on simple motor tasks (78), it is possible that age contributed to motor slowing in our chronic pain group. Our analysis did not probe whether age was a factor in these differences, but this should be considered in subsequent studies using alternative analytical techniques.

To our knowledge, this is the first study to evaluate a scoring system that focuses on maximum pain attenuation during a task as a measure of distraction effects on chronic pain. A distraction effect was characterized by finding an association between better performance on the tasks and a reduction in chronic pain. Firstly, it was unexpected that few people exhibited a reduction in their pain intensity and unpleasantness and that changes in chronic pain in both dimensions were not related to task engagement in the hard task. Only a small portion of people (*n* = 5) exhibited clinically relevant pain intensity modulation (i.e., reduction of ≥30%) in the simple task, and no individuals experienced clinically relevant pain intensity reduction during the hard task. McCaul and Malott (16) indicate that for a task to effectively reduce pain, it should not be controlled and automatic. A controlled and automatic task would not be engaging enough to effectively capture a person's attention away from pain. Contrarily, we found that engagement in a simple motor task could reduce some individuals' pain intensity, sometimes even to a clinically relevant level. We further found that those with greater task engagement in the simple task had a higher per cent reduction in pain intensity. However, it was unexpected that our data from the hard task were not in line with other studies that found engagement in more attentionally demanding tasks can reduce the perception of experimentally induced pain (36, 37, 48–50, 63, 81). Given that we found a significant association between faster RTs and chronic pain intensity reduction in the simple task, we believe that a distraction effect was captured by the PAT scoring system. Since this association did not exist in the hard task, the scoring system was likely capturing natural fluctuations of chronic pain.

One caveat to note is that we did not counterbalance the order of the tasks, which could have contributed to the lack of significant findings in the hard task. However, this does not exclude the possibility that temporary chronic pain relief through distraction can occur for some individuals during an automated motor activity, based on our findings of clinically relevant decreases in pain intensity in a portion of individuals (*n* = 5) and a significant relationship between task engagement (mean RTs) and chronic pain intensity reduction. Our findings suggest that distraction may not be an effective pain-coping strategy for everyone with chronic pain but could be effective for a small subset of individuals for short-lasting chronic pain relief. These findings also echo an earlier call for identifying specific individuals who may benefit from certain attention-based pain management techniques for chronic pain (43). Unfortunately, our small sample size is a limitation of the study that hampered our ability to identify distinguishing characteristics in those who experienced clinically relevant changes to their chronic pain. Furthermore, since we included individuals with low average chronic pain intensity over the last 4 weeks (<3/10), it is possible that their low levels of pain were not attention-grabbing enough to demonstrate the effects of distraction as a pain-coping strategy through the PAT system. A sample with a larger distribution of average pain levels would enable the evaluation of the PAT effect across individuals with varying baseline trait chronic pain.

Attention is not a unitary cognitive process but rather encompasses many sub-types. Thus, the attentional resources engaged to complete a specific task may not be shared with the attentional resources used to attend to chronic pain. Torta et al. (21) noted that there are different attentional processes (82, 83) that can modulate pain (e.g., selective attention, divided attention, voluntary vs. involuntary attention, internal vs. external attention, etc.). As a task becomes more complex, it may invoke multiple aspects of attention, some of which may differ from those associated with chronic pain. There have been other attentionally demanding tasks that have significantly reduced experimental pain in healthy people (36), but it is not known whether the attentional mechanisms underlying this effect are similar to those used in the context of chronic pain. Furthermore, there were no significant relationships between the other task engagement metrics (RT SD and % Acc) and measurements of maximum change in chronic pain intensity or unpleasantness in both tasks. The absence of significant findings could be related to the choice of attention-demanding task for the paradigm. Additionally, there was overall high group accuracy in the hard task. It is possible that our hard task was not challenging enough to elicit a distraction effect, but that would contradict our findings of a significant task engagement effect on chronic pain reduction in our simple motor task. Nevertheless, other tasks that show more variation in performance across all task engagement metrics (RT, RT SD, and % Acc) should be explored to examine the relationship between task engagement and chronic pain across a wider spectrum of performance levels. It is worth exploring whether these relationships exist in all the intended measurements of task engagement using other attentionally demanding tasks that have shown success in healthy people (36) and that are potentially more difficult than our current hard task.

The lack of correlation between the maximum change in pain and task engagement in the hard task may also be impacted by our study design. For example, the hard task was presented after the easier tasks, and so some participants may have been fatigued at this point. Vogel et al. (49) demonstrated that cognitive fatigue can reduce performance on a task and subsequently the pain attenuation experienced for experimentally induced pain. Additionally, an issue noted in other studies of distraction is that rating pain during a task creates a paradoxical attention to pain (25, 30, 84). This sort of divided attention effect could have been induced in the current study because participants knew that they would be providing chronic pain ratings after each block and may have periodically attended to their pain during the task. Furthermore, in healthy people, motivation, interest, and the value placed on a task (26) are important factors in prioritizing attention between pain and task performance (85). The motivation, value, and interest a participant has towards a task may influence whether the task leads to strong enough attentional engagement to result in an analgesic effect. Here, we did not evaluate the effect of incentivization or moderate motivation, value, or interest towards task performance on the numeric card tasks, but it is possible that these factors contributed to the distraction effect (or lack thereof in most of the sample) in the chronic pain group.

As part of our supplemental exploratory analysis, we found differences in pain-related characteristics between PAT sub-groups for pain intensity on the simple and hard tasks. In both tasks, the negative PAT group (pain increase during the task) had the highest group-level score on the painDETECT, indicating that this group had a higher likelihood of having neuropathic pain. Attributes of pain that increase its novelty and salience, like spontaneous and paroxysmal characteristics in neuropathic pain, can be attention-demanding and interfere with cognitive processes (2). In the negative PAT group, the attention-demanding qualities of neuropathic pain possibly captured their attention and increased their perception of their chronic pain during the task. This may have impeded their ability to experience pain attenuation through task engagement. This novel finding could reflect preliminary evidence of differences in the effectiveness of pain coping by distraction based on chronic pain types (i.e., neuropathic vs. non-neuropathic pain); however, we acknowledge that the sample size was quite small in each PAT sub-group. Given the small sample size, these results should be taken with caution but can reflect a proof-of-concept of potential pain-related differences in PAT sub-groups. Although the effect sizes for this analysis were large, there is nonetheless a need for a larger sample size to validate and increase the reliability of the results. Unfortunately, we could not further sub-divide the sample into other pain types other than neuropathic pain due to the limited sample size.

In conclusion, this study introduces the PAT scoring system, which shows potential for capturing individual differences in the distraction effect on chronic pain. Although we observed some people experiencing changes in their chronic pain to a clinically relevant degree, overall, our data suggest that distraction may not be an effective long-term behavioural coping strategy for chronic pain relief. Furthermore, prolonged avoidance approaches to chronic pain could lead to increased distress and suffering (86). Instead, coping methods that enhance acceptance of chronic pain and psychological flexibility may be more effective alternatives for chronic pain relief (86, 87). Nevertheless, our work shows that even an automated, simple task can be valuable for characterizing the distraction effect in people with chronic pain and serve as a simpler, translatable clinical tool. Since our PAT system was applied to relatively short tasks, future studies are needed to examine persistence in longer duration attention-demanding tasks that allow for greater temporal variations in pain to occur. Hence, future validation studies are needed for this PAT scoring system to determine whether it can identify individuals who may benefit from components of attention-based pain management approaches, such as distraction, that are used to manage chronic pain.

## Data Availability

The datasets presented in this article are not readily available because all requests for data must be approved by the institution (note that study participants include patients). Requests to access the datasets should be directed to Karen Davis, karen.davis@uhn.ca.
