# Supplementary material for: Pain attenuation during task: a proof-of-concept study for a new scoring metric to capture individual differences in chronic pain changes by task engagement
Source: Front Pain Res (Lausanne). 2026 Oct 26;7:1945745. doi: 10.3389/fpain.2026.1945745 (PMC13745498; doi:10.3389/fpain.2026.1945745)
Supplement: Supplementary file 1 [file Datasheet1.pdf]

Pain attenuation during task: a proof-of-concept study for a new scoring metric to capture individual differences in chronic pain changes by task engagement

Vaidhehi Veena Sanmugananthan, Rima El-Sayed, Robert D Inman, and Karen Deborah Davis

<u>Supplementary Material (Figures)</u>

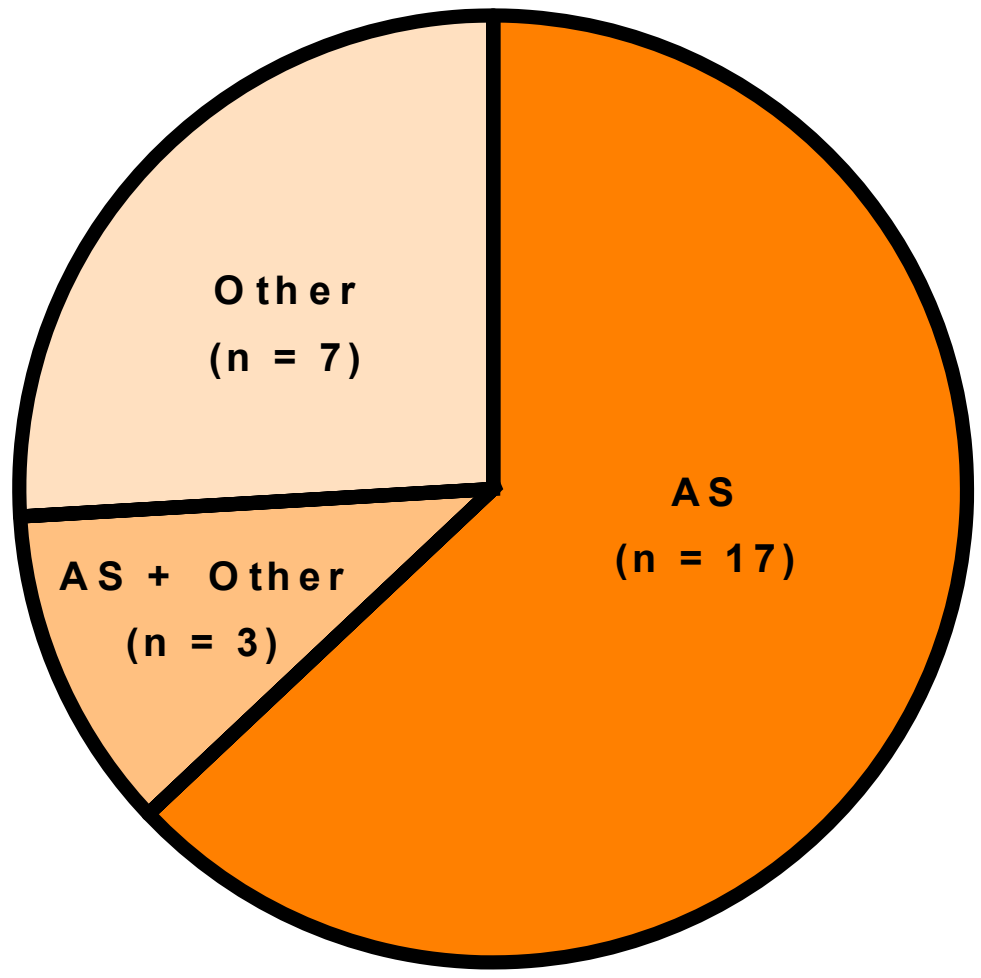

Supplementary Figure 1. Pain conditions

In total, twenty-seven individuals with chronic pain were involved in this study. Seventeen people had only ankylosing spondylitis (AS), three people had AS & other pain conditions, and seven had pain conditions other than AS. The other pain conditions reported by these participants included: pain in different body areas that were not AS-related (e.g. foot, shoulder, knee, fingers, neck), chronic back pain not AS-related, disc degeneration, sciatica, arthritis, Crohn's disease, irritable bowel syndrome, non-infectious osteomyelitis, migraines, s-shaped scoliosis, and/or temporomandibular joint pain.

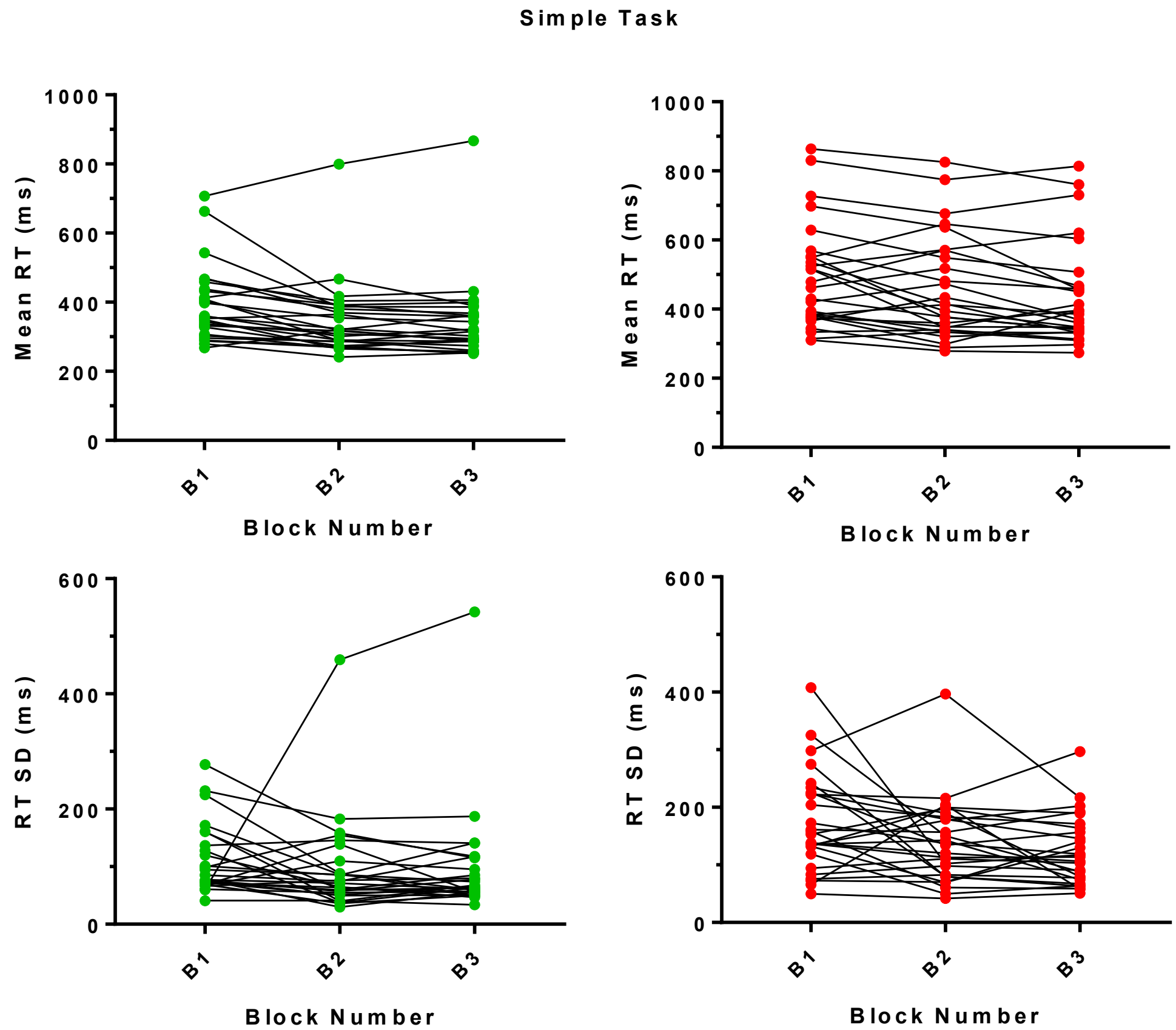

Supplementary Figure 2. Within-subject reaction times across blocks in the simple motor task

The within-subject mean reaction time (RT) and RT standard deviation (SD) within each block in the simple task are shown for the healthy controls (HCs) and people with chronic pain. The green circles represent the HCs and the red circles represent the chronic pain group.

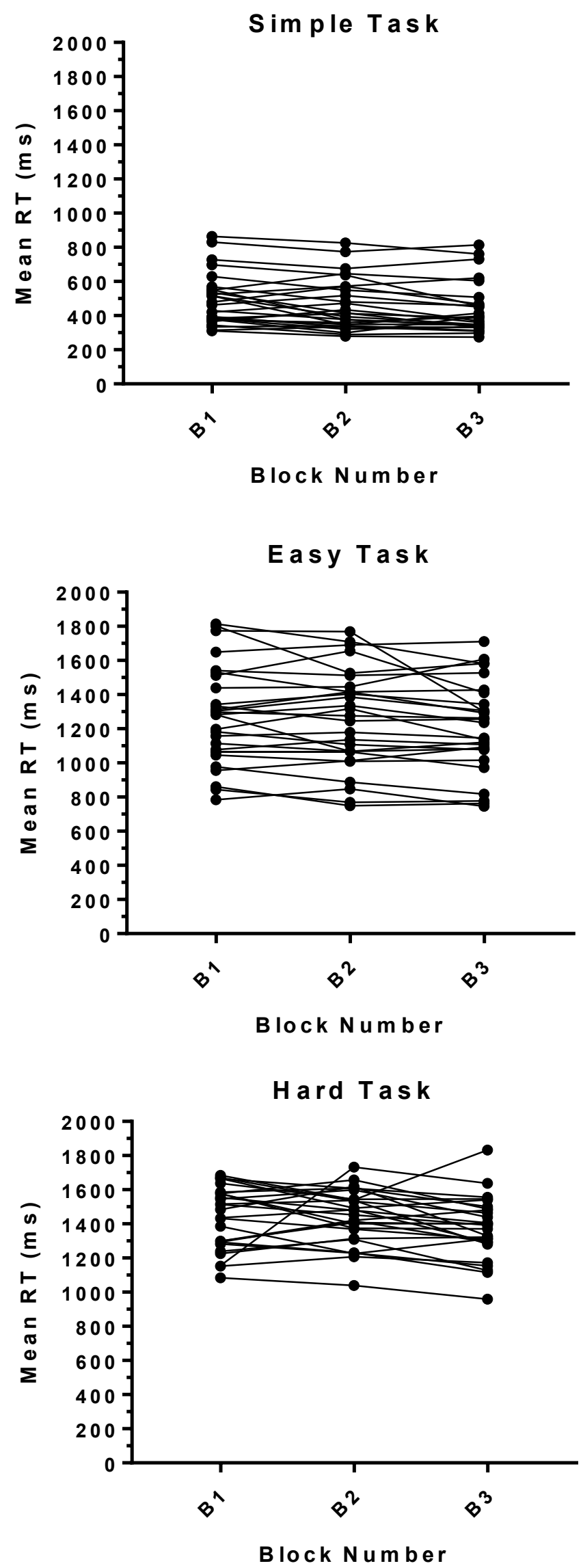

Supplementary Figure 3. Within-subject reaction times across blocks in all tasks

The within-subject mean reaction times (RT) in each block for all tasks (simple, easy and hard) are shown for the people with chronic pain.

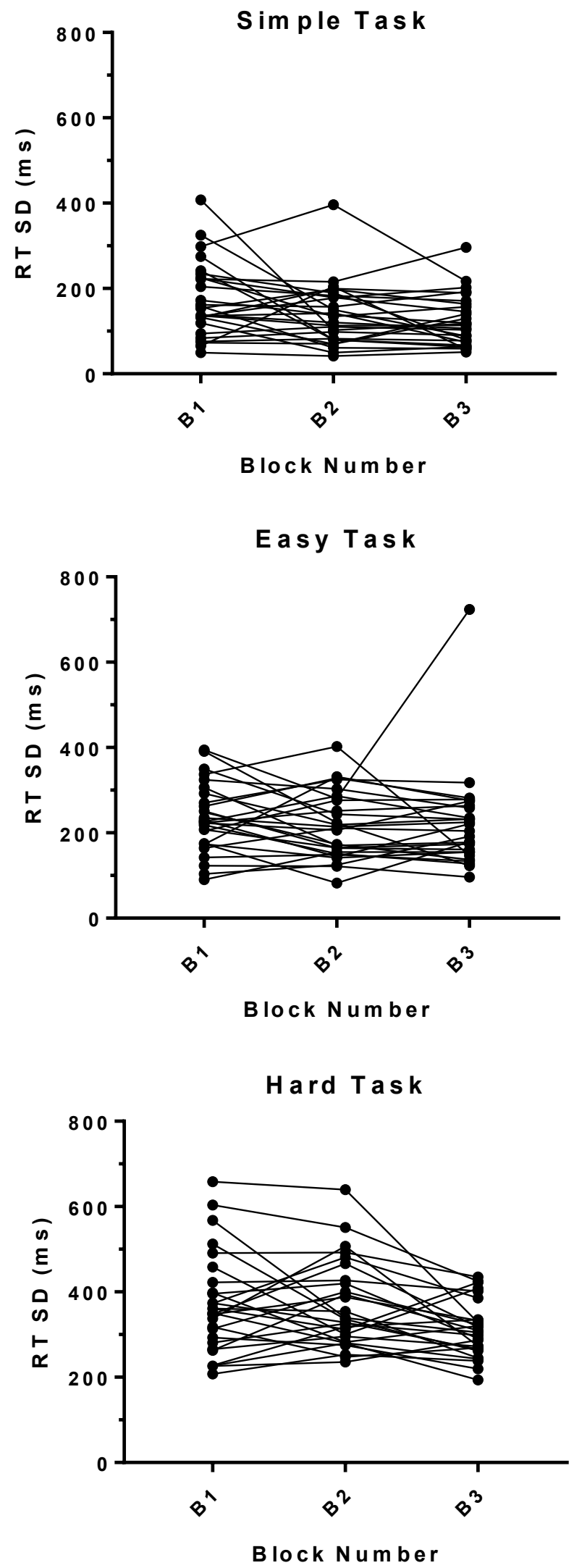

Supplementary Figure 4. Within-subject reaction time standard deviations across blocks in all tasks

The within-subject reaction time standard deviation (RT SD) in each block for all tasks (simple, easy and hard) are shown for the people with chronic pain.

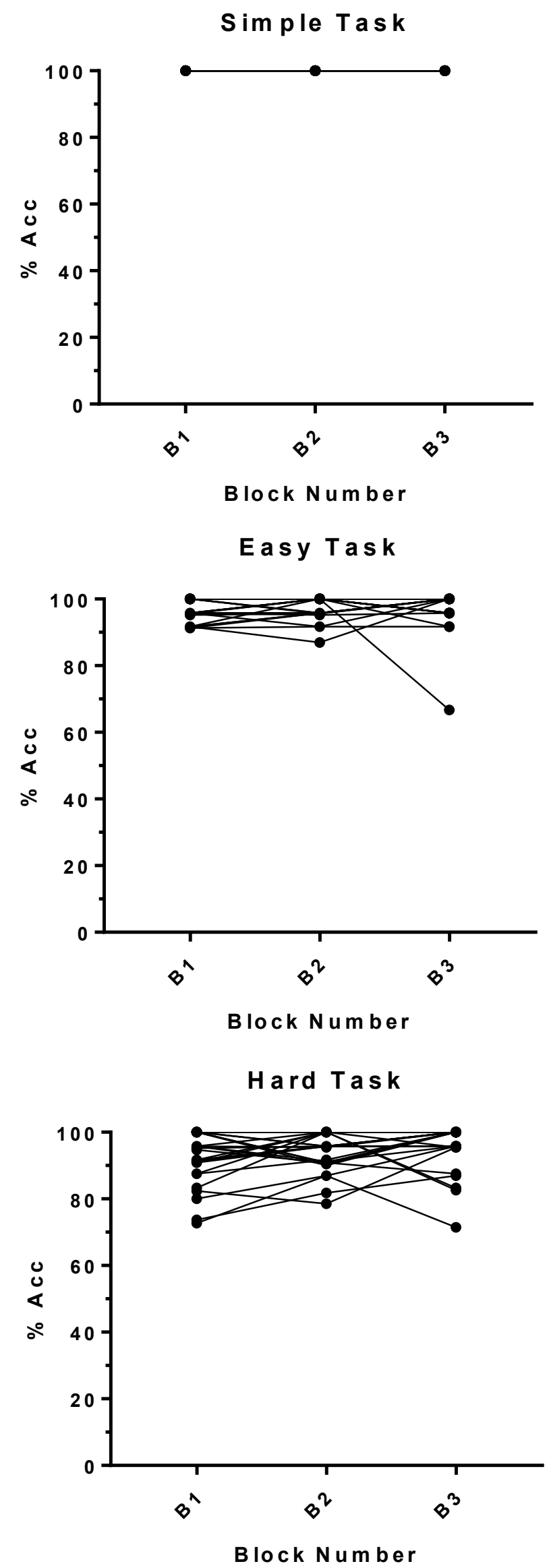

Supplementary Figure 5. Within-subject percent accuracy across blocks in all tasks

The within-subject percent accuracy (% Acc) in each block for all tasks (simple, easy and hard) are shown for the people with chronic pain.

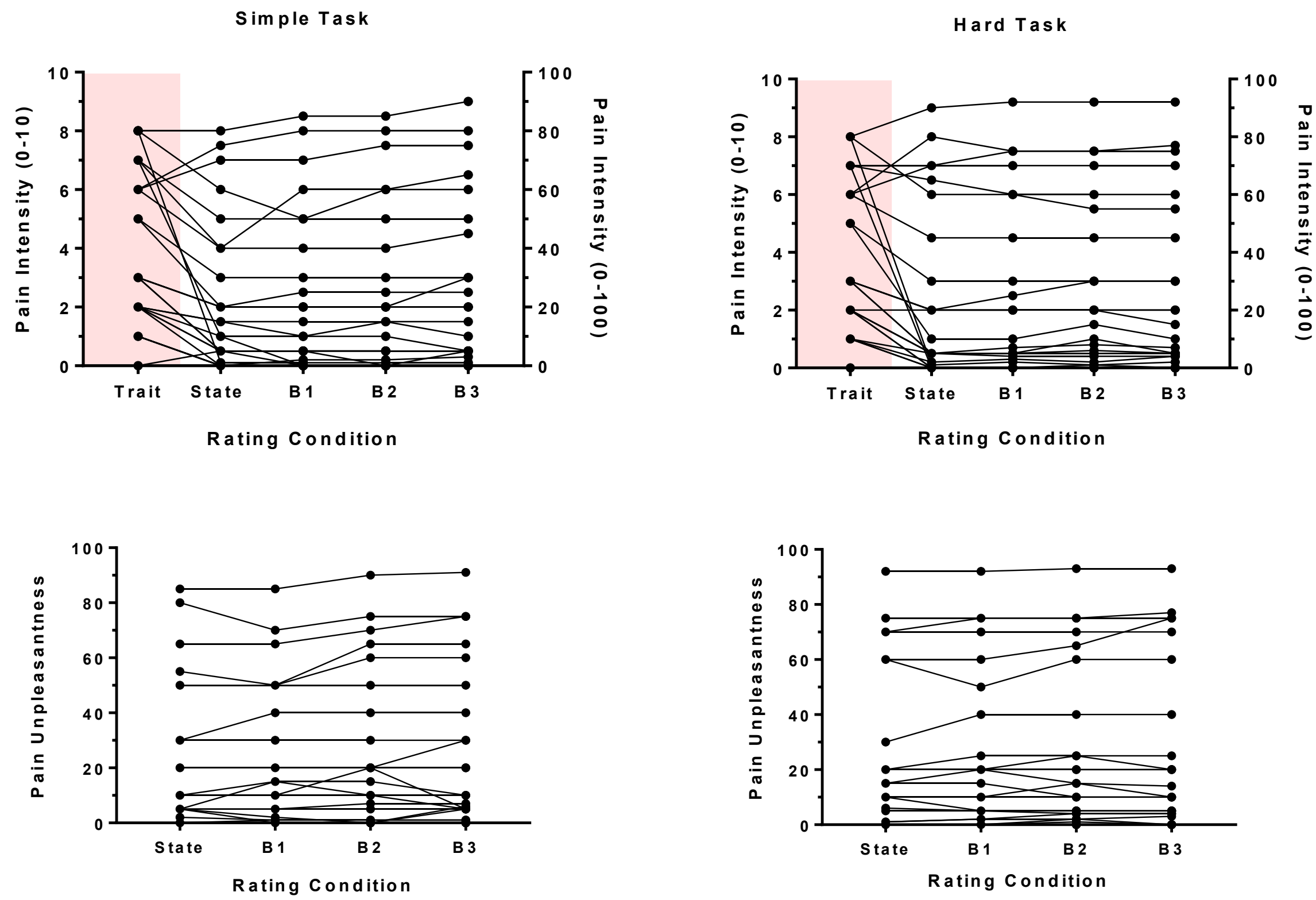

Supplementary Figure 6. Chronic pain ratings in simple and hard tasks

The within-subject baseline state (pre-task) and post-task block ratings for pain intensity and unpleasantness (0 - no pain/not unpleasant at all, 100 – worst pain imaginable/ most unpleasant imaginable) are shown for the simple and hard tasks. The pain intensity graphs include baseline trait pain ratings inside the red box (average pain in the past 4 weeks taken from painDETECT questionnaire; 0 – no pain, 10 – max pain) to showcase trait and state pain intensity.
